# Supplementary material for: CattleNet-XAI: An explainable CNN framework for efficient cattle weight estimation
Source: PLoS One. 2025 Nov 13;20(11):e0336434. doi: 10.1371/journal.pone.0336434 (PMC12614548; doi:10.1371/journal.pone.0336434)
Supplement: Attachment — Submitted filename: Response to Reviewers.pdf [file pone.0336434.s002.pdf]

Response to Reviewers

Manuscript Number: PONE-D-25-09685

Title: CattleNet-XAI: An Explainable CNN Framework for Efficient Cattle Weight Estimation

The authors would like to thank the editor and reviewers for their constructive comments and suggestions that have helped improve the quality of this manuscript. The manuscript has undergone a thorough revision according to the reviewers' comments. Please see our responses below (Pages 1–6 for Reviewer-1 and Page 7 for Reviewer-2). For the reviewers' convenience, we have made significant changes in the revised manuscript and highlighted in Yellow.

Reviewer 1

Reviewer Point #1 — Table 2 compares MAE values with studies using much smaller datasets (e.g., 20–34 cattle). The authors should contextualize these comparisons, as their larger dataset (513 cattle) may inherently enable better performance. How do the author's account for dataset size differences when comparing performance? Were additional metrics (e.g., MAPE) considered for a fairer comparison? The manuscript compares MAE with what authors refer to as "recent previous" papers. I have two concerns here. Metrics like MAE or RMSE can be influenced by the scale of the data, and MAPE is often preferred when comparing predictive models across different studies. RMSE is the most commonly used indicator of predictive performance and I have no issues with using it to compare models using the same dataset. It's the use of metrics like MAE and RMSE for comparison between studies that I have issues with. If a study used smaller cows on average (due to any reason such as the breed or the age of sampled cows, the farm situation, etc), it will surely result in lower RMSE a priori compared to a study that used on average larger cows. In addition, the comparisons need to include most recent publications as the research area is rapidly advancing. For instance, recent papers such as those of Gebreyesus et al. (https://doi.org/10.3389/fgene.2022.947176) and Hou et al (https://doi.org/10.1016/j.compag.2023.108184), both from 2023, show lower MAPE values.

Reply: We thank the reviewer for highlighting this important point. In response, we have revised the manuscript to improve the fairness and clarity of our cross-study comparisons. **Inclusion of MAPE for Fairer Cross-Study Comparisons:** To ensure that our comparisons are robust across studies involving cattle of varying breeds, sizes, and weight distributions, we have included Mean Absolute Percentage Error (MAPE) as an additional evaluation metric. Unlike MAE and RMSE, which are sensitive to scale, MAPE provides a normalized, scale-independent measure of error. This allows for more meaningful performance comparisons between studies that may involve different weight ranges or cattle types. The revised **(Table**

1) now includes MAPE values for all models, and we have updated the Results section accordingly to reflect and interpret these values in line 648. It is observed that the 3Conv3Dense model achieved a MAPE of approximately 6.22%.

Reviewer Point # 2 — Prediction of body weight The system implemented here might be considered an overkill. Body weight have been shown to be one of the easiest traits to predict from images given the high correlation between dimension (captured from images/pixel size) and body weight across species. Several studies show the possibility of BW prediction with simpler setups such as a single overhead camera and simpler models. The system proposed here makes sense only if additional value is taken out of it such as the prediction of other, more challenging conditions like lameness or stress responses. Given the authors claim to address the need for high throughput and easier measuring system, why go for complex models?

Reply: Thank you for the valuable comment. We agree that body weight is often considered one of the easier traits to predict due to its high correlation with body dimensions. However, our study focused on using real-world 2D cattle images. In such cases, using a CNN-based approach allows the model to learn complex visual patterns beyond basic height and width. While simpler models like linear regression and random forest performed reasonably well, our results clearly show that our custom CNN architecture (3Conv3Dense) significantly outperformed them as well as the more complex pre-trained EfficientNetB3, which is implemented in our revised version. For example, our CNN achieved a lower MAE (18.02 kg) compared to EfficientNetB3 (21.05 kg), despite being lighter and more tailored to our specific task. Additionally, we used LIME to explain CNN's predictions, which highlighted regions like the rib cage and abdomen as key contributors to the model's decisions. This explainability is crucial for trust and transparency in real-world use. Our model offers a balanced approach as it is more robust than basic regressors, simpler than heavy models like EfficientNetB3, and still efficient enough for practical deployment. While predicting weight was our main goal, the proposed system is designed to be scalable for more complex traits like lameness detection in the future.

Reviewer Point # 3 — High-throughput? The authors claim that their method addresses the need for high-throughput cattle weight estimation. High-throughput applications in real-world farm settings involve multiple animals, occlusions, and complex environments (e.g., fences, farm equipment, varied lighting conditions). he dataset used in this study appears to consist of controlled images without clear discussion of occlusions or real-world production settings.

Were images captured in open farm environments, or were they taken under controlled conditions?

Does the model account for partial occlusions (e.g., cattle overlapping, fences blocking parts of the body)?

How does the model handle low-resolution or motion-blurred images, which are common in farm settings?

Reply: We thank the reviewer for raising this important point regarding the real-world applicability of our system in high-throughput farm environments. Our current dataset consists primarily of side-view images of individual cattle, many of which were taken in relatively

controlled settings with minimal occlusion. We acknowledge that this does not fully represent the complexity of real-world farm scenarios, where animals may overlap, lighting may vary, and motion blur or background clutter can impact image quality. At this stage, our primary goal was to develop a proof-of-concept model that could accurately predict cattle weight using unconstrained, side-view images without requiring physical measurements or fixed-camera setups. However, we have incorporated histogram equalization and normalization to improve contrast and robustness to lighting variability. In future work, we plan to expand the dataset to include images from operational farms, featuring occlusions, multiple animals, varying lighting, and different angles. We have now revised the manuscript to clearly state the limitations of our current dataset and added a discussion on our future plans for real-world deployment.

Reviewer Point # 4 — Novelty & justification of approach The study introduces CNN-based cattle weight estimation, but similar approaches have been explored (e.g., EfficientNet-based livestock weight prediction, Transformer-based vision models). What specific advancements does CattleNet-XAI offer over prior deep learning models? A more in-depth comparison with state-of-the-art methods (not just RF and LR) is needed.

Reply: Thank you for the valuable feedback. Our primary contribution lies in integrating LIME-based interpretability with a custom CNN model, which we refer to as CattleNet-XAI. This combination provides not only accurate cattle weight predictions but also model transparency which enables insights into which regions of the images influence predictions, which is critical for trust and adoption in agricultural sectors. While prior works have explored deep learning models like EfficientNet and transformer-based architectures for livestock analysis, few have emphasized explainability tailored to end-user needs. In response to the reviewer's suggestion, we have additionally implemented the EfficientNetB3 model highlighted in line number 469 in our revised manuscript and incorporated its results into our comparative analysis [Table-1]. This allows for a deeper evaluation of CattleNet-XAI against a state-of-the-art baseline, further validating its performance and practical relevance.

Reviewer Point # 5 — Model interpretability (explainable AI - XAI) The paper claims to use LIME (Local Interpretable Model-agnostic Explanations) for explainability, but there is insufficient analysis. What were the most influential features in LIME? Were certain image regions consistently emphasized in weight predictions? Could SHAP (SHapley Additive Explanations) be a better alternative to LIME in this case?

Reply: We appreciate the reviewer's insightful question regarding model interpretability. LIME was used to visualize which parts of the image contributed most to the predicted weight. Our analysis showed that LIME consistently highlighted regions such as the rib cage, hindquarters, and abdomen areas that are logically correlated with muscle and fat mass, thus aligning well with visual weight assessments used by livestock experts. We agree that SHAP is a powerful alternative for explainability, particularly in structured/tabular data or when model internals are accessible. However, since our pipeline involves CNNs trained on raw image inputs, LIME was chosen for its flexibility in image-based local interpretability. Nonetheless, in future work, we plan to incorporate Deep SHAP or Grad-CAM to provide complementary global explanations at the feature map level. We also update our revised manuscript in that manner in line number 859.

Reviewer Point # 6 — The dataset contains 17,899 images, but only 2052 are original images. The impact of synthetic images or augmentations on model performance and bias needs discussion. How diverse are the cattle breeds, and do model errors differ by breed, age, or body size? Can this model generalize to different environments, lighting conditions, or angles? The dataset includes 513 unique cattle with multiple images per individual. Without clear details on how the data were split (e.g., ensuring no overlap of the same cattle in training and test sets), there is a high risk of data leakage, which could artificially inflate model performance. This is a major scientific flaw that undermines the validity of the results. How were the images of the same cattle partitioned across training, validation, and test sets? Were subject-wise splits used to ensure independence? The authors apply normalization before histogram equalization, which is unconventional. Typically, histogram equalization is performed first to enhance contrast, followed by normalization. This sequence could affect feature extraction and model performance. Question: Why was this order chosen? Was there empirical evidence supporting this approach? If not, consider revising the pipeline. The dataset is heavily skewed toward the LOCAL breed (68.8%), with underrepresented breeds like BRAHMA and MIR KADIM (0.4% each). This raises concerns about the model's generalizability to other breeds. Were techniques like stratified sampling, data augmentation, or weighted loss functions used to address class imbalance? If not, how do the authors justify the model's applicability to underrepresented breeds?

Reply: In this study, we applied image normalization prior to histogram equalization, deviating from the conventional order. Specifically, pixel values were first scaled to the [0, 1] range to standardize the intensity distribution across images. This step reduces the impact of lighting variability and prepares the data for consistent histogram operations. After normalization, the image was converted back to an 8-bit format to enable channel-wise histogram equalization, enhancing local contrast in a controlled manner. Finally, the image was re-normalized to [0, 1] for input into the CNN. This ordering was found to improve training stability and validation performance, particularly by preventing over-enhancement in underexposed regions. Although the dataset contains an imbalanced distribution of cattle breeds, we did not apply oversampling techniques, as the model's predictions are primarily based on body features such as the rib cage, abdomen, and overall body shape characteristics and that remain largely consistent across different breeds. While there are some breed-specific differences in traits like color, head shape, or horn structure, as discussed in our XAI section (line 859), these factors have minimal influence on weight estimation in our model.

We may analyze breed-wise prediction accuracy. In the future, we will evaluate the model's performance by breed to improve and validate our approach.

Reviewer Point # 7 — Comparison with advanced architectures The study primarily compares the proposed CNN model to Random Forest and Linear Regression, which are known to be weaker for image-based tasks. How would CattleNet-XAI compare against pretrained vision models like ResNet, EfficientNet, or Vision Transformers (ViTs)? Were pre-trained networks (transfer learning) considered? If not, why? The authors use YOLOv5 for feature extraction but do not justify why this version was chosen over newer alternatives (e.g., YOLOv8) or other object detection models. Additionally, the number of features selected via Recursive Feature Elimination (RFE) and their interpretability are not discussed. Question: Why was YOLOv5 chosen? How many features

were selected via RFE, and what were the most important features for weight prediction?

Reply: In response to your suggestions, we expanded our study by implementing the EfficientNetB3 model and including its results in our comparative analysis. Deatils are descrived in line number 469. also it's results with other graphs such as validation curves, predicted graph or residual graphs are also discussed in each section.

We selected YOLOv5 for object-centric feature extraction due to its strong trade-off between speed and accuracy, along with ease of integration. Although newer versions like YOLOv8 are available, YOLOv5 was effective and stable at the time of our experiments. All the reasoning are discussed in our revised menuscript from line number 313 in our Feature Extraction using YOLOv5 section.

RFE was used to select the most predictive features from the high-dimensional CNN feature space. While RFE helps reduce model complexity and improve accuracy, it does not directly offer anatomical interpretability. To address this, we employed LIME-based visual explanations, which consistently highlighted regions such as the rib cage, abdomen, and hindquarters as contributing most to the model's predictions. This combination of feature selection and visual interpretation confirms that our model leverages semantically meaningful features for cattle weight estimation. we have included everything in our revised manuscript in line number 341.

Reviewer Point # 8 — Hyperparameter tuning optimization

The paper does not describe how hyperparameters (e.g., learning rate, batch size, number of filters, dropout rates) were selected. Was any hyperparameter tuning done (e.g., grid search, Bayesian optimization)? Without tuning, the performance gains of the CNN model may not be fully optimized.

The authors propose a 3Conv3Dense CNN but do not provide a thorough justification for this architecture. Why were three convolutional layers and three dense layers chosen? Were other architectures (e.g., ResNet, EfficientNet) explored?

Reply: Thank You for your comment. We have revised the manuscript to provide a more detailed explanation of our hyperparameter tuning and architectural decisions (Section 3.4: Hyperparameter Tuning and Optimization in line number 532). Specifically, we performed empirical tuning of key hyperparameters, including learning rate, batch size, dropout rate, and L2 regularization. While we did not use automated search techniques such as grid search or Bayesian optimization due to computational constraints, we iteratively tested a range of values informed by prior work and deep learning best practices. The final configuration (learning rate = 0.0001, batch size = 40, dropout = 0.03, L2 = 0.001) consistently delivered strong generalization with low MAE and MAPE across multiple trials. Regarding architectural choices, we evaluated four different custom CNN architectures with varying depths of convolutional and dense layers such as 2Conv2Dense, 2Conv3Dense, 3Conv2Dense, and 3Conv3Dense. Our results demonstrated that the 3Conv3Dense model achieved the best predictive accuracy. We did explore EfficientNetB3 via transfer learning, as mentioned in Section 3.3, but found that our tailored architecture, combined with optimized preprocessing and tuning, yielded superior performance for our dataset and task.

Reviewer Point # 9 — Overfitting and generalization issues The validation loss curves indicate some degree of overfitting, especially in shallower CNN architectures. Was

dropout or regularization (L1/L2) used to mitigate overfitting? Did the model undergo k-fold cross-validation, or was only a single train-test split used? The paper lacks details on cross-validation, train-test split ratios, and hyperparameter tuning. Without this information, reproducibility is compromised. What were the train-test split ratios? Were hyperparameters tuned using cross-validation? If so, what were the optimal values?

Reply: Thank you for your valuable feedback. In response, we would like to clarify that dropout layers and L2 regularization were applied in our deep learning models to mitigate overfitting. Additionally, the dataset was split into 70% for training, 20% for testing, and 10% for validation. We also incorporated 10-fold cross-validation to ensure robust evaluation and improve the reliability of our results. These methodological details, along with information on hyperparameter tuning, have been clearly stated in the revised manuscript to support reproducibility and transparency. Data splitations are discussed in line number 357 and for hyperparameter a new section is added in our revised menuscript in line numbe 532.

Reviewer Point # 10 —

Regression model feature engineering The Recursive Feature Elimination (RFE) process is mentioned but lacks sufficient detail. What features were selected as most important? Would feature extraction via Principal Component Analysis (PCA) improve the performance of regression models?

Reply: Thank your for your comment. Revised and detailed are explained in our revised manuscript as per reviewer comment also included details RFE and feature seleection from line number 341.

Reviewer 2

Reviewer Point # 1 —

Review This study presents a CNN built for estimating cattle weight. The results show a relevant improvement in the method's accuracy compared to the traditional random forest and linear regression methods. The study is relevant since it offers immediately applicable advantages.

Comments: The writing is very clear and grammatically correct. Different methods are presented educationally, thus, this paper could serve as a quick reference tool for the field of traditional machine learning algorithms. At some passages, however, the writing seems exaggerated. For example, the last line in the Abstract says "presenting a revolutionary method for cattle management". In the conclusions, it reads "The model's exceptional performance underscores the promise of deep learning in automating agricultural activities, diminishing dependence on manual and error-prone weight estimation techniques. This innovation could improve precision agriculture by providing a scalable, non-invasive method for real-time livestock management." This excessive flourish tone is not coherent with an objective interpretation of the results of an experiment or the evaluation of a method. I think the inappropriateness of this style may not reflect the authors' spirit about their work. Perhaps this results from using an automatic writing polishing tool, which tends to push beyond convenient limits, the impact the text will create. This is only my explanation, and it may well be pure speculation. But the fact is that this paper's content compares several techniques that existed dozens of years ago.

Presenting figures next to their caption makes reviewing this manuscript difficult. Please put the Figures in their place.

Final comments: This paper presents an interesting application of Convolutional Neural Networks. The manuscript is well organized, but some processing components as Figures and Tables, must be finished and located where the readers can better observe them. I believe it can be improved considerably by just objectively sticking to the results. I would reconsider this manuscript for publication if an improved version is presented.

Reply: Thank you for your valuable comments. We have carefully reviewed each point and have revised the manuscript accordingly. For instance, we modified the abstract's wording as per your suggestion and also adjusted other sentences throughout the text based on the reviewers' feedback.
