# Supplementary material for: CattleNet-XAI: An explainable CNN framework for efficient cattle weight estimation
Source: PLoS One. 2025 Nov 13;20(11):e0336434. doi: 10.1371/journal.pone.0336434 (PMC12614548; doi:10.1371/journal.pone.0336434)
Supplement: Attachment — Submitted filename: Response_to_Reviewers_R2.pdf [file pone.0336434.s003.pdf]

Response to Reviewers

Manuscript Number: PONE-D-25-09685R2

Title: CattleNet-XAI: An Explainable CNN Framework for Efficient Cattle Weight Estimation

The authors would like to thank the editor and reviewers for their constructive comments and suggestions that have helped improve the quality of this manuscript. The manuscript has undergone a thorough revision according to the reviewers' comments. Please see our responses below (Pages 1 for Reviewer-2 and Page 2-4 for Reviewer-3). For the reviewers' convenience, we have made significant changes in the revised manuscript and highlighted in Yellow.

Reviewer 1

No comment was found from Reviewer 1 during this review feedback from the journal.

Reviewer 2

Reviewer Point # 1 —

Some of my comments in the former review were addressed. No doubt the manuscript has improved; The writing is clean, and the structure of the document, together with the completeness of the types of neural networks included in the tests, makes this study a valid consultation document. However, I feel a lack of novelty in the study that could be considered conflicting with some assertions made at the end of the document. In the conclusions, for example, the document says:

"The model's exceptional performance underscores the promise of deep learning in automating agricultural activities, diminishing dependence on manual and error-prone weight estimation techniques. This innovation could improve precision agriculture by providing a scalable, non-invasive method for real-time livestock management."

When the reader intends to identify which is the model that has EXCEPTIONAL performance, the reader realizes this is a difficult task. Perhaps that is because the subject of that exceptionality does not exist in the document. At least I do not see a model proposed in the study. Finally, the Section Conclusions do not show a conclusion. Instead, a vague text rich in rhetoric that, in my opinion, does not fit well in the conclusions segment of a scientific publication.

Reply: Thank you for your valuable feedback. We have made updates based on your suggestions, including rewriting the entire Conclusion and Future Work sections (in line number 920). Additionally, we have highlighted our novelty in both the abstract and introduction sections in line number 67.

Reviewer 3

Reviewer Point #1 —
The paper lacks in major contribution and motivation of research.

Reply:
We thank the reviewer for their feedback. Our research aims to solve the main problems with how cattle weight is currently measured. Existing methods are either too expensive, like 3D systems , or they are "black box" AI models that farmers don't trust because it's unclear how they work. Our major contribution is CattleNet-XAI framework, which presents a novel, cost-effective solution using 2D images. This framework features a custom "3Conv3Dense" CNN that achieves a state-of-the-art Mean Absolute Error of 18.02 kg, significantly outperforming prior work. Critically, its novelty lies in the integration of Explainable AI (XAI) using LIME, which provides visual proof that the model bases its predictions on anatomically relevant features like the rib cage and abdomen. By being both highly accurate and transparent, our method helps build the trust needed for AI in agriculture, which is a significant and practicle. And all of these contributions are explicitly listed at the end of the Introduction (Line no 67) in our manuscript

Reviewer Point #2 —
The background and significance of this study should be highlighted in the abstract.

Reply: Thank you for your comment. We have revised the abstract according to your comment. We have added the background of the study and clearly explained its significance in our revised abstract.

Reviewer Point #3 — Check the English presentation of this paper to remove the typo mistakes. Some grammatical issues need to be addressed in the whole text. Please reform the long paragraphs. Please polish the writing and English of the manuscript carefully. The writing of the paper needs a lot of improvement in terms of grammar, spelling, and presentation. The paper needs careful English polishing since there are many typos and poorly written sentences. I found several errors.

Reply: We sincerely thank the reviewer for their valuable feedback. In response, we have gone through the entire text to correct all grammatical errors and typos (like in line number 80, 109, 140, 192, 241, 284 etc.). We have also restructured sentences and paragraphs to improve clarity, flow, and overall readability. And all the changes are marked as yellow in our revised manuscript.

Reviewer Point #4 —
In the "Introduction" section, a more detailed analysis of the existing literature on the subject is needed, and an in-depth analysis of the possible application fields.

Reply:

The entire introduction has been carefully reviewed. Although we have a separate literature review section still based on your feedback, we have updated the introduction section accordingly and added a short paragraph (lines 28–42) discussing existing work on this subject along with its potential applications.

Reviewer Point # 5 —

The mathematics used throughout the article is still not very strict. Please try to update and illustrate some elements in the mathematical model that are not defined very strictly.

Reply: We have gone through each of the mathematics updated where needed also we have added three new equations for Dropout Rate (in line number 554), L2 Regularization (in line number 557) and R^2 value (in line number 639).

Reviewer Point # 6 —

The overall structure of the article should be improved.

Reply: We appreciate the reviewer's suggestion to improve the article's structure. In response, we have carefully revised the manuscript to enhance this. We focused on strengthening the narrative in the introduction as well as conclusion. Also improved the transitions between sections, and better organizing the Result Analysis section with clearer subheadings to guide the reader. We believe these revisions have made the paper's structure much stronger and easier to follow.

Reviewer Point # 7 —

The result part is week, results and discussion should be better explained.

Reply: We sincerely thank the reviewer for this constructive feedback. We agree that the Results and Discussion section required more depth and have revised it extensively to better explain our findings.

The updated section (from line number: 641) now provides a more detailed description of what each performance metric signifies in a practical context. We have also expanded the comparative analysis to better explain why our proposed model outperformed the baselines, discussing the impact of architectural choices and the advantages of a domain-specific model.

Reviewer Point # 8 —

References must be updated and add the suitable from the following https://doi.org/10.3390/en12050961, https://doi.org/10.1109/EIConRus.2018.8317170, https://doi.org/10.1016/j.est.2024.113556, https://doi.org/10.1109/ACCESS.2024.3437191, https://doi.org/10.1109/MEPCON.2017.8301313, https://dx.doi.org/10.21608/jaet.2021.82231, https://doi.org/10.1109/MEPCON47431.2019.9008171, https://doi.org/10.21608/sej.2021.155557, https://doi.org/10.1109/MEPCON58725.2023.10462371, DOI: 10.1109/ACCESS.2024.3525183, https://doi.org/10.1007/s00521-024-09433-3, https://doi.org/10.1371/journal.pone.0317619, https://doi.org/10.20508/ijrer.v14i2.14346.g8898,
https://doi.org/10.20508/ijrer.v13i1.13718.g8659, https://doi.org/10.1007/s00521-024-09433-3, https://doi.org/10.1007/s00521-024-09902-9,
https://doi.org/10.21608/SVUSRC.2024.279389.1198,

Reply:

Thank you for your suggestions. We found that two of the suggested papers are relevant to our work and have cited them at lines 526 and 630.

Reviewer Point # 9 —

Check all of your Figures and Tables have a good explanation of your text.

Reply: Thank you for your comment. We have checked and updated extensively where needed like we have updated Fig-6 caption in line number 389. Also Figure -13 caption in line number 393.

Reviewer Point # 10 —

Many paragraphs without citations

Reply: We have carefully reviewed the entire article and added citations where necessary to better support our claims and methods.

Reviewer Point # 11 —

What are the contributions and novelty of work mentioned?

Reply: The primary contribution and novelty of this research are consolidated in CattleNet-XAI, a framework that introduces a high-performance, explainable, and rigorously validated solution for cattle weight estimation. The core of this work is the development of a custom 3Conv3Dense CNN architecture, which is a novel, lightweight model tailored specifically for this weight prediction task.

We address the critical "black box" issue in agricultural AI by integrating Explainable AI (XAI) using LIME visualizations. This provides transparent, trustworthy evidence that the model's predictions are based on anatomically correct regions like the rib cage and abdomen, enhancing its practical value. All of this is highlighted in the introduction section of our manuscript, specifically at line 67.

Reviewer Point # 12 —

The authors' conclusions need to be improved, a comparison of the results obtained with those already existing in the literature would be appropriate. I suggest also describing what can still be improved in this work, which can still be improved based on the results obtained, according to the authors' view. It is suggested to offer some limitations existed in this study and an outlook for future study in the last section.

Reply:

Thank you for your comment. We have thoroughly rewritten the entire Conclusion and Future Work sections. Additionally, we already included a Limitations (Line 885) section where we described the constraints of this study. A comparison table (Table 2, Line 840) is also provided to contrast our work with existing studies.
