# Supplementary material for: CattleNet-XAI: An explainable CNN framework for efficient cattle weight estimation
Source: PLoS One. 2025 Nov 13;20(11):e0336434. doi: 10.1371/journal.pone.0336434 (PMC12614548; doi:10.1371/journal.pone.0336434)
Supplement: Attachment — Submitted filename: 2025.09.Clatle.pdf [file pone.0336434.s004.pdf]

CatleNet-XAI: An Explainable CNN Framework for Efficient Cattle Weight Estimation

2nd review

During the first and second reviews of this paper, I presented numerous criticisms about this study. The subject of the study is well supported and shows a valid aspect of reality deserving the application of CNN Models, thus, most of the problems presented in my reviews could be qualified as formal issues.

The authors have addressed the points I referred to in previous inspections. Including my concern about the lack of supported results of the study. In its current condition, the manuscript presents conclusions that are consistent with the quantitative results sustained in the text. The statistical assessment of the method is now well presented, and the formal aspects of mathematics is clearly shown in the text. In my opinion, this manuscript may now be published.
