# Supplementary material for: CattleNet-XAI: An explainable CNN framework for efficient cattle weight estimation
Source: PLoS One. 2025 Nov 13;20(11):e0336434. doi: 10.1371/journal.pone.0336434 (PMC12614548; doi:10.1371/journal.pone.0336434)
Supplement: Attachment — Submitted filename: Cow_PlosOne_Journal_Response.pdf [file pone.0336434.s005.pdf]

Response to Reviewers

Manuscript Number: PONE-D-25-09685R3

Title: CattleNet-XAI: An Explainable CNN Framework for Efficient Cattle Weight Estimation

The authors would like to thank the editor and reviewers for their constructive comments and suggestions that have helped improve the quality of this manuscript. The manuscript has undergone a thorough revision according to the reviewers' comments. Please see our responses below (Pages 1 for Reviewer-2 and Page 2-3 for Reviewer-4). For the reviewers' convenience, we have made significant changes in the revised manuscript and highlighted in Yellow.

Reviewer 1

During this revision, no comment from Reviewer-1.

Reviewer 2

Reviewer Point # 1 —

During the first and second reviews of this paper, I presented numerous criticisms about this study. The subject of the study is well supported and shows a valid aspect of reality deserving the application of CNN Models, thus, most of the problems presented in my reviews could be qualified as formal issues.

The authors have addressed the points I referred to in previous inspections. Including my concern about the lack of supported results of the study. In its current condition, the manuscript presents conclusions that are consistent with the quantitative results sustained in the text. The statistical assessment of the method is now well presented, and the formal aspects of mathematics is clearly shown in the text. In my opinion, this manuscript may now be published.

Reply: Thank you for your valuable feedback. We have made updates based on your suggestions, including rewriting the entire Conclusion and Future Work sections. Additionally, we have highlighted our novelty in both the abstract and introduction.

Reviewer 3

During this revision, no comment from Reviewer-3.

Reviewer 4

Reviewer Point # 1 — Lack of Novelty: The manuscript claims to present a novel framework, but upon review, the methodology closely follows existing techniques without introducing significant advancements. While the use of CNNs and XAI (Explainable AI) in livestock weight estimation is a relevant and growing field, the paper does not sufficiently differentiate itself from prior works. The proposed CNN model, though effective, lacks a clear distinction from other models already well-established in the literature. The contribution, as described, does not present sufficient novelty to justify publication.

Reply:
We thank the reviewer for raising this important point. We acknowledge that CNNs and XAI have been applied in related domains; however, our work introduces several aspects that differentiate it from prior studies:

- Customized CNN Architecture: We designed and evaluated multiple lightweight custom CNN models (3Conv3Dense), specifically optimized for cattle image data, achieving an MAE of 18.02 kg, which is lower than previously reported results in the literature.
- Dual-Method Framework: Our study is one of the first to compare two fundamentally different approaches, end-to-end CNN training and YOLOv5-based feature extraction with regression, offering new insights into the trade-offs between deep learning and traditional ML for cattle weight estimation.
- Integration of XAI (LIME) in Livestock Weight Prediction: While explainability has been explored in medical imaging, to our knowledge, this is the first application in cattle weight estimation. Our results highlight which body regions (rib cage, abdomen, hindquarters) most influence predictions, bridging the trust gap for practical adoption.
- Error Analysis and Practical Relevance: In addition to prediction metrics, we provide residual/error analysis, offering practical insights into conditions where models perform suboptimally, a dimension often overlooked in existing work.

We have updated our contribution section in line number 77 and 89 and marked as yellow in our revised manuscript.

Reviewer Point # 2 — Methodological Weakness: The explanation of the methodology is not sufficiently rigorous. For instance, the handling of the dataset, feature extraction techniques, and model performance evaluation need clearer justifications and explanations. The manuscript mentions advanced techniques like LIME for model interpretability but does not provide a comprehensive evaluation of how these methods are integrated and their actual impact on improving model transparency.

Reply: We appreciate the reviewer's concern. We would like to clarify that the methodology section already includes details on dataset handling (collection, preprocessing steps such as image-to-array conversion, normalization, and histogram equalization, as well as the 70/10/20 split), feature extraction and selection (YOLOv5 with RFE), and performance evaluation (MAE, MSE, RMSE, MAPE, R^2, and cross-validation), along with the hyperparameter settings

used. We also integrated LIME to highlight biologically meaningful body regions (rib cage, abdomen, hindquarters), thereby demonstrating model transparency.

Reviewer Point # 3 —

Unclear Results and Evaluation: While the paper presents results for the proposed models, the analysis is superficial. The comparison between different models (CNNs, EfficientNetB3, and traditional methods like Random Forest) is not sufficiently detailed, especially in terms of real-world applicability. A more thorough discussion on the limitations of the current model and areas for further improvement is needed.

Reply: Thank you for your feedback. We appreciate the opportunity to clarify where this information is located in our manuscript, as it was indeed present in the original submission.

A detailed comparison of the models real-world applicability is located in our "Result Analysis" section line no 646. We specifically discuss the trade-off between the superior accuracy of our proposed CNN (3Conv3Dense) (MAE of 18.02 kg) and its significantly higher computational efficiency (11,430 ms training time) compared to other methods.

Also, the manuscript contains dedicated sections for a thorough discussion of the model's shortcomings ("Limitations") and areas for improvement ("Conclusion and Future Work"). These sections detail the dataset's geographical limitations and our specific plans for a breed-specific performance analysis.

Reviewer Point # 4 — Lack of Clear Conclusion: The conclusions drawn from the results are vague and lack specificity. The statement that the proposed model "could improve precision agriculture" seems speculative and not supported by a strong, comparative analysis of existing literature and methodologies. The absence of concrete future directions or a critical evaluation of the model's shortcomings makes the conclusion less impactful.

Reply: Thank you. We have reviewed the entire manuscript and updated the conclusion accordingly (line 943) by adding a direct quantitative comparison, acknowledging the model's limitations, and specifying concrete future directions

Reviewer Point # 5 — Writing and Grammar Issues: While some revisions were made, the manuscript still contains numerous grammatical errors and poorly constructed sentences. Several sections remain unclear, and the overall readability of the paper is compromised. A more thorough proofreading and restructuring are necessary. In conclusion, while the topic is of potential interest, the manuscript lacks sufficient novelty, rigorous methodology, and a thorough analysis of results to merit publication. I recommend rejecting the paper and suggesting that the authors reconsider their approach, including refining the model's uniqueness, addressing the methodological gaps, and providing a more robust discussion of their findings.

Reply: We thank the reviewer for this observation. We have carefully gone through the manuscript line by line and revised it accordingly, correcting grammatical errors, improving sentence structure, and enhancing overall readability (line no : 49, 63, 832 898, 907). These updates ensure that the manuscript is clearer, more polished, and easier to follow.
