# Supplementary material for: CattleNet-XAI: An explainable CNN framework for efficient cattle weight estimation
Source: PLoS One. 2025 Nov 13;20(11):e0336434. doi: 10.1371/journal.pone.0336434 (PMC12614548; doi:10.1371/journal.pone.0336434)
Supplement: Attachment — Submitted filename: 2025.04.CattleWeightEstim.Review.docx [file pone.0336434.s001.docx]

**CattleNet-XAI: An Explainable CNN Framework for Efficient Cattle Weight Estimation**

**Review**

This study presents a CNN built for estimating cattle weight. The results show a relevant improvement in the method's accuracy compared to the traditional random forest and linear regression methods. The study is relevant since it offers immediately applicable advantages.

**Comments:**

The writing is very clear and grammatically correct. Different methods are presented educationally, thus, this paper could serve as a quick reference tool for the field of traditional machine learning algorithms.

At some passages, however, the writing seems exaggerated. For example, the last line in the Abstract says “presenting a revolutionary method for cattle management”. In the conclusions, it reads “The model’s exceptional performance underscores the promise of deep learning in automating agricultural activities, diminishing dependence on manual and error-prone weight estimation techniques. This innovation could improve precision agriculture by providing a scalable, non-invasive method for real-time livestock management.” This excessive flourish tone is not coherent with an objective interpretation of the results of an experiment or the evaluation of a method. I think the inappropriateness of this style may not reflect the authors’ spirit about their work. Perhaps this results from using an automatic writing polishing tool, which tends to push beyond convenient limits, the impact the text will create. This is only my explanation, and it may well be pure speculation. But the fact is that this paper’s content compares several techniques that existed dozens of years ago.

Presenting figures next to their caption makes reviewing this manuscript difficult. Please put the Figures in their place.

**Final comments:**

This paper presents an interesting application of Convolutional Neural Networks. The manuscript is well organized, but some processing components as Figures and Tables, must be finished and located where the readers can better observe them. I believe it can be improved considerably by just objectively sticking to the results. I would reconsider this manuscript for publication if an improved version is presented.
